# Targetless Radar–Camera Calibration via Trajectory Alignment

**DOI:** 10.3390/s25247574

**Published:** 2025-12-13

**Authors:** Ozan Durmaz, Hakan Cevikalp

**Affiliations:** Electrical and Electronics Engineering Department, Eskisehir Osmangazi University, Meselik, Eskisehir 26040, Turkey; hakan.cevikalp@gmail.com

**Keywords:** radar, camera, sensor calibration, trajectory alignment, synchronization

## Abstract

Accurate extrinsic calibration between radar and camera sensors is essential for reliable multi-modal perception in robotics and autonomous navigation. Traditional calibration methods often rely on artificial targets such as checkerboards or corner reflectors, which can be impractical in dynamic or large-scale environments. This study presents a fully targetless calibration framework that estimates the rigid spatial transformation between radar and camera coordinate frames by aligning their observed trajectories of a moving object. The proposed method integrates You Only Look Once version 5 (YOLOv5)-based 3D object localization for the camera stream with Density-Based Spatial Clustering of Applications with Noise (DBSCAN) and Random Sample Consensus (RANSAC) filtering for sparse and noisy radar measurements. A passive temporal synchronization technique, based on Root Mean Square Error (RMSE) minimization, corrects timestamp offsets without requiring hardware triggers. Rigid transformation parameters are computed using Kabsch and Umeyama algorithms, ensuring robust alignment even under millimeter-wave (mmWave) radar sparsity and measurement bias. The framework is experimentally validated in an indoor OptiTrack-equipped laboratory using a Skydio 2 drone as the dynamic target. Results demonstrate sub-degree rotational accuracy and decimeter-level translational error (approximately 0.12–0.27 m depending on the metric), with successful generalization to unseen motion trajectories. The findings highlight the method’s applicability for real-world autonomous systems requiring practical, markerless multi-sensor calibration.

## 1. Introduction

The fusion of heterogeneous sensors has become central to robust perception in autonomous mobile robotic systems. Vision-based Simultaneous Localization and Mapping (SLAM) provides rich structure and semantics but degrade under adverse conditions, motivating complementary sensing and cross-modal fusion [1,2]. Early systems emphasized cameras and Light Detection and Ranging (LiDAR) sensors as primary modalities and addressed their mutual calibration and timing issues in automotive settings [3]. In this context, millimeter-wave (mmWave) radar offers complementary strengths —operability in rain, fog, dust, and direct sunlight, resilience to illumination changes, and Doppler velocity measurements—making radar–camera fusion an attractive path to resilient perception [4,5]. Practical multi-sensor platforms increasingly consider radar alongside vision and LiDAR, leading to tools and formulations for extrinsic calibration that explicitly include radar, camera, and LiDAR, and to robustness-focused radar calibration methods for intelligent transportation scenarios [6,7].

Concurrently, deep learning has reshaped radar perception. Networks have improved object detection and fusion throughput (e.g., You Only Look Once (YOLO) variants), and have been studied for radar–vision integration at scale [8,9]. For extrinsics, both target-based and targetless strategies have been investigated. Target-based co-calibration with dedicated artifacts can achieve high precision but is cumbersome outside controlled environments [10]. Targetless alternatives automatically exploit natural motion and scene dynamics across multiple 3D sensors (lidars and radars) [11], rotational auto-calibration for radar–camera pairs [12], continuous-time optimization for 3D radar-to-camera alignment [13], online multi-sensor calibration via moving-object tracking [14], and spatiotemporal radar–camera calibration methods [15].

Several targetless radar–camera calibration methods have been proposed in recent years. Schöller et al. [12] introduced a planar-motion approach that estimates rotational alignment using 2D radar scans. Jin et al. [16] extended motion-based calibration to automotive radars but still required relatively dense point clouds. Du et al. [17] employed a multi-step geometric pipeline combining radar and vision features but reported large translation errors, especially along the Z-axis. More recently, Zhu et al. [18] used trajectory-driven event association for roadside radar–camera calibration and provided a comprehensive accuracy benchmark.

Recent targetless radar–camera calibration approaches generally fall into three categories: (1) continuous-time spatiotemporal optimization [13,15], (2) motion-based auto-calibration relying on 3D structure and trajectory consistency [11,12], and (3) learning-based extrinsic estimation from radar–vision pairs [14,19]. Continuous-time formulations achieve high accuracy but require computationally expensive nonlinear optimization and dense radar returns, which limits applicability to automotive radars. Motion-based methods assume stable, well-observed trajectories and are sensitive to radar sparsity or low radar cross-section (RCS) conditions. Learning-based approaches deliver strong performance but require large annotated datasets and high-end Graphics Processing Units (GPUs), making them difficult to deploy in robotics platforms.

Despite this progress, radar–camera calibration remains challenging. Radar returns are sparse, noisy, and intermittent due to radar cross-section variability; therefore, robust preprocessing is essential, including density-based clustering, outlier rejection, and careful temporal synchronization to avoid geometry-altering misalignment [20,21,22].

Beyond classical geometric and motion-based calibration approaches, recent research has increasingly explored deep learning-based radar–camera extrinsic estimation. These methods typically rely on convolutional or Siamese network architectures that learn feature correspondences between radar tensors and camera images. For example, Ibrahim et al. [23] provide a comprehensive review of deep learning pipelines such as CalibNet, LCCNet, and RegNet, highlighting their dependence on dense automotive radar returns and large annotated datasets. Similarly, the Sensors study of 2024 [24] reports sub-centimeter translation accuracy and sub-degree rotation errors on KITTI-style ground-vehicle trajectories using deep neural networks trained end-to-end.

While these approaches demonstrate strong performance in structured outdoor driving environments, they are not directly applicable to our setting. Deep learning models require dense radar point clouds or range–azimuth–Doppler tensors, whereas mmWave radars observing small aerial targets yield extremely sparse returns (3–10 points per frame). Moreover, UAV motion exhibits full 6-DoF dynamics rather than the largely planar motion assumed in automotive datasets. Finally, these networks require extensive labeled datasets and high computational cost for training, none of which exist for indoor UAV radar–camera calibration. In contrast, the trajectory-alignment method proposed in this work requires no training data, remains robust under sparse measurements, and is fully targetless, making it well suited for low-RCS aerial robotics applications.

This work proposes a fully targetless radar–camera extrinsic calibration method that aligns trajectories of a moving target observed by the radar and the camera [25,26]. After preprocessing, we estimate the rigid transformation via closed-form solvers that minimize alignment error over matched 3D points [27,28]. Accuracy is quantified against high-precision ground truth from an OptiTrack motion capture system [29]. While recent learning-based radar detectors (e.g., RadDet and YOLO-ORE) advance radar perception, they typically require substantial training resources; our pipeline intentionally favors lightweight, noise-robust processing to remain deployable in constrained settings [19,30]. For vision, we employ YOLOv5 for drone detection and integrate depth to recover 3D positions; timestamps and data streams are handled in Robot Operating System 2 (ROS 2) Humble to ensure consistent logging and reproducibility [31,32]. Our formulation follows standard rigid pose estimation principles and practices surveyed broadly in the pose-estimation literature [33].

The main contributions of this work are summarized as follows:

- **Targetless radar–camera calibration:** A complete trajectory-alignment framework that eliminates dedicated targets, suitable for indoor/outdoor and dynamic scenarios where pattern placement is impractical.
- **Noise-robust preprocessing:** A dual-stream pipeline—YOLOv5-based 3D localization for the camera; DBSCAN clustering and RANSAC filtering for sparse radar returns—paired with passive Root Mean Square Error (RMSE)-based temporal synchronization to handle timestamp offsets without hardware triggers [20,22,31,32].
- **Closed-form extrinsics:** Rigid transformation estimation using Kabsch and Umeyama solvers, yielding consistent accuracy under realistic radar noise characteristics [27,28].
- **Ground-truth validation and generalization:** Evaluation against OptiTrack ground truth with cross-trajectory tests confirming transferability of the estimated extrinsics [29].
- **Practical design choice:** In contrast to more resource-intensive learning pipelines for radar (e.g., RadDet, YOLO-ORE), our design emphasizes simplicity and portability while retaining robustness [19,30].

Compared to prior targetless approaches, our method integrates modern detection, density-based clustering, passive synchronization, and closed-form alignment into a single pipeline validated with high-precision ground truth. The result is improved robustness to radar sparsity and systematic range bias, while preserving the simplicity required for field deployment.

## 2. Methodology

This section describes the complete pipeline of the proposed targetless radar–camera extrinsic calibration method. The process begins with the integration of the mmWave radar and RGB-D camera into a synchronized experimental setup, followed by the collection of simultaneous measurements of a moving drone. Raw sensor data are preprocessed to remove noise and extract reliable 3D trajectories, using YOLOv5-based detection for the camera stream and DBSCAN clustering with RANSAC filtering for the radar stream. The trajectories are then temporally synchronized through passive RMSE-based time offset estimation, ensuring frame-to-frame correspondence between the two modalities. Finally, the spatial relationship between the radar and camera coordinate frames is determined by estimating a rigid transformation via Kabsch and Umeyama [27,28] algorithms. The accuracy and robustness of the estimated transformation are validated against high-precision ground truth measurements obtained from an OptiTrack motion capture system.

### 2.1. Sensor Integration and Experimental Setup

The experimental setup consisted of two primary sensors: a Texas Instruments IWR1843 mmWave radar (Texas Instruments, Dallas, TX, USA) and an Intel RealSense D435i red–green–blue plus depth (RGB-D) camera (Intel Corporation, Santa Clara, CA, USA). Both were mounted on stable tripods placed approximately 2 m apart, facing a common flight volume. The sensors were intentionally positioned at a moderate angular offset so that their fields of view partially overlapped, simulating realistic multi-sensor mounting conditions in robotic platforms.

The dynamic target was a Skydio 2 drone (Skydio, Inc., Redwood City, CA, USA), chosen for its ability to perform smooth and repeatable flight trajectories. Its onboard stabilization and autonomous navigation allowed consistent motion patterns, making it suitable for trajectory-based calibration.

To obtain high-precision ground truth, an OptiTrack motion capture system (NaturalPoint, Inc., Corvallis, OR, USA) equipped with four Flex 3 infrared cameras surrounded the flight area. Retroreflective marker clusters were rigidly attached to the radar, camera, and drone, enabling precise 3D tracking at 240 Hz with sub-millimeter accuracy. Markers were mounted using lightweight rigid frames to prevent vibration or relative displacement during experiments.

**Figure 1:**
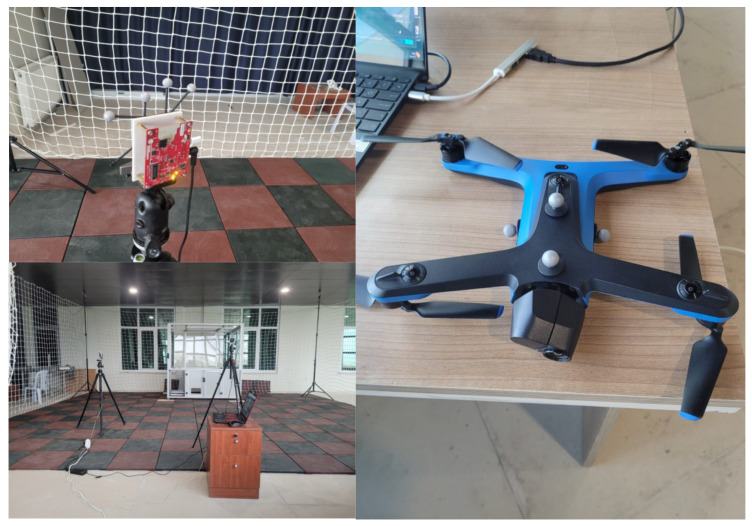
Experimental setup and equipment. (**Top left**): radar module; (**bottom left**): indoor area; (**right**): Skydio 2 drone.

The indoor laboratory was arranged to minimize occlusions and ensure stable lighting. The flight volume was enclosed with a safety net for operational safety. This controlled setup enabled synchronized and reliable data collection from all three systems—radar, camera, and OptiTrack. An overview of the sensor placement, experimental environment, and the Skydio 2 drone are shown in Figure 1.

### 2.2. Radar and Camera Data Processing

For data processing, we utilized three main algorithms: YOLOv5 [31], DBSCAN [20], and RANSAC [21]. These algorithms are summarized below.

#### 2.2.1. YOLOv5

You Only Look Once version 5 (YOLOv5) is a single-stage, deep learning-based object detection framework that directly predicts bounding box coordinates and class probabilities from an input image in a single forward pass [31]. It employs a Cross-Stage Partial Darknet (CSP-Darknet) backbone for feature extraction, a Path Aggregation Network (PANet) for multi-scale fusion, and anchor-based detection heads for localization and classification. In this study, YOLOv5 was custom-trained to detect the Skydio 2 drone in RGB images captured by the camera. The model outputs 2D bounding boxes, which are associated with aligned depth maps to estimate 3D drone positions in the camera coordinate frame. Detections with confidence scores below 0.5 were discarded to avoid unreliable estimates. YOLOv5’s robustness to lighting and pose variations ensured consistent performance during dynamic flights.

#### 2.2.2. DBSCAN

DBSCAN (Density-Based Spatial Clustering of Applications with Noise) is an unsupervised clustering algorithm that groups points into dense regions using two parameters: ε (maximum neighborhood radius) and MinPts (minimum number of points required to form a cluster) [20]. Unlike centroid-based clustering (e.g., k-means), DBSCAN detects clusters of arbitrary shape and identifies sparse points as noise, making it well-suited for radar point clouds that are often irregular and noisy.

In our method, DBSCAN was applied to each radar frame to isolate the cluster corresponding to the drone’s radar reflection. We empirically set ε=0.5 m and MinPts = 3 to balance detection robustness and false positives. If no valid cluster was found—for instance, due to low radar cross-section or temporary dropouts—a fallback centroid of all retained radar points was computed to maintain trajectory continuity.

#### 2.2.3. RANSAC

RANSAC (Random Sample Consensus) is a robust model-fitting algorithm designed to estimate model parameters in the presence of outliers [21]. It iteratively samples subsets, fits a model, and identifies inliers that agree within a tolerance threshold. In this study, RANSAC was applied to both radar and camera trajectories to reject spurious detections. Points with residuals greater than a spatial threshold τ=0.3 m were removed as outliers.

#### 2.2.4. Notation

Let $t_{i}$ and $t_{j}$ denote timestamps of the camera and radar data streams, respectively. At each radar frame $t_{j}$, the raw point cloud is represented as $P_{r}\left(t_{j}\right)={\left\{p_{r}^{\left(k\right)}\in R^{3}\right\}}_{k=1}^{|P_{r}\left(t_{j}\right)|}$, where $p_{r}^{\left(k\right)}$ is the *k*-th 3D radar return and $|P_{r}\left(t_{j}\right)|$ is the number of detected points. For the camera, $P_{c}\left(t_{i}\right)\in R^{3}$ denotes the 3D drone position obtained from YOLOv5 detection and depth projection. A hat symbol $\left(\hat{\cdot }\right)$ indicates a filtered or estimated value, while a bar $\left(\bar{\cdot }\right)$ denotes an average (centroid). The Euclidean norm is $∥x∥=\sqrt{x^{T}x}$.

#### 2.2.5. Data Processing Pipeline

Camera-based processing began with YOLOv5 detections on RGB frames from the camera. The centroid of each bounding box was back-projected into 3D using the corresponding depth map, forming a discrete trajectory $\left\{P_{c}\left(t_{i}\right)\right\}$. A moving-average filter smoothed short-term fluctuations while preserving motion dynamics. For a window size w=5, the smoothed position $\hat{P}\left(t_{i}\right)$ was computed as:

(1) $$\hat{P}\left(t_{i}\right)=\frac{1}{w}\sum_{j=i-\lfloor w/2\rfloor }^{i+\lfloor w/2\rfloor }P\left(t_{j}\right)$$

This low-pass filter attenuated jitter caused by detection noise, while confidence filtering (>0.5) excluded uncertain samples.

Radar data required more extensive preprocessing. Due to the drone’s low radar cross-section and composite body, the radar produced sparse and inconsistent returns. Each radar frame $P_{r}\left(t_{j}\right)$ was first constrained to the known flight volume. Remaining detections were clustered using DBSCAN with ε=0.5 m and MinPts = 3. Clusters satisfying |Cluster|≥MinPts were retained, and their centroids provided position estimates. If clustering failed, a fallback geometric centroid was used:

(2) $${\bar{p}}_{r}\left(t_{j}\right)=\frac{1}{|P_{r}\left(t_{j}\right)|}\sum_{k=1}^{|P_{r}\left(t_{j}\right)|}p_{r}^{\left(k\right)}$$

Although this introduces uncertainty, it maintains trajectory continuity and prevents null frames. After clustering, both radar and camera trajectories underwent outlier rejection using RANSAC-based local filtering. Within a temporal window, a linear motion model was fit and residuals were computed as:

(3) $$r_{i}=∥P\left(t_{i}\right)-\hat{P}\left(t_{i}\right)∥$$

Points with $r_{i}>\tau$ (typically τ=0.3 m) were discarded. This effectively removed spurious radar points and mislocalized YOLO detections. Finally, the filtered radar and camera trajectories were interpolated onto a unified time grid:

(4) $$P\left(t\right)=P\left(t_{k}\right)+\frac{t-t_{k}}{t_{k+1}-t_{k}}\;\left(P\left(t_{k+1}\right)-P\left(t_{k}\right)\right)$$

This ensured temporally aligned and spatially smoothed trajectories, providing synchronized input for subsequent RMSE-based temporal alignment and rigid transformation estimation.

### 2.3. Temporal Synchronization

Temporal misalignment between radar and camera measurements is a common challenge in multi-sensor systems, particularly when sensors are triggered asynchronously or lack hardware-level synchronization. Even small offsets in timestamps can lead to significant spatial errors during trajectory alignment. To address this issue, we implemented a passive temporal synchronization approach inspired by Olson’s method [22], which estimates the time offset by minimizing geometric discrepancies between corresponding trajectories.

Let $P_{r}\left(t\right)\in R^{3}$ denote the 3D position of the detected object (i.e., the drone) at time *t* as measured by the radar, and $P_{c}\left(t\right)\in R^{3}$ the corresponding position from the camera. Due to the temporal misalignment Δt, the measurements are not time-matched, such that:

(5) $$P_{r}\left(t+\Delta t\right)\approx P_{c}\left(t\right)$$

To estimate the optimal Δt, a grid search was conducted within a predefined temporal window Δt∈[−2s,+2s] with a step size of δ=0.01s. For each candidate offset, radar points were linearly interpolated in time to generate a time-matched trajectory ${\hat{P}}_{r}\left(t\right)$, and the Root Mean Square Error (RMSE) between the radar and camera trajectories was computed as:

(6) $$\mathrm{RMSE}\left(\Delta t\right)=\sqrt{\frac{1}{N}\sum_{i=1}^{N}{∥{\hat{P}}_{r}\;\left(t_{i}+\Delta t\right)-P_{c}\left(t_{i}\right)∥}^{2}}$$

where *N* is the number of synchronized time samples. The value of Δt that minimizes RMSE was selected as the optimal temporal alignment. This method requires no prior assumptions about motion models and is robust to noise and sparse data, making it suitable for radar–camera calibration where measurements may be irregular or asynchronous.

Intuitively, a temporal offset directly manifests as a spatial offset along the drone’s path. Let p(t) denote the true 3D position of the drone at time *t*. The camera observes $p_{c}\left(t\right)\approx p\left(t\right)$, while the radar effectively reports $p_{r}\left(t\right)\approx p\left(t+\Delta t\right)$ due to processing and buffering delays. For a drone moving with instantaneous velocity $v\left(t\right)=\dot{p}\left(t\right)$, the resulting displacement between the two measurements can be approximated as:

(7) ∥p(t+Δt)−p(t)∥ ≈ ∥v(t)∥·|Δt|

With typical flight speeds of 1–1.5 m/s in our experiments, the estimated offset of Δt=0.46 s therefore corresponds to a spatial shift of roughly 0.5–0.7 m along the drone’s path. This magnitude is consistent with the order of the spatial discrepancy observed between radar and camera trajectories before temporal alignment.Note that this temporal-offset-induced shift is not directly visible in the final aligned figures, since they already depict the trajectories after applying the optimal Δt and the rigid-body transform.

Although the radar and camera trajectories lie in different coordinate frames and do not spatially overlap before calibration, they encode the same temporal motion pattern: turning points, curvature changes, and velocity peaks occur at the same relative times in both streams. Temporal offset estimation thus does not require prior knowledge of the rigid transformation. Once the optimal Δt is found and applied, corresponding radar and camera samples refer to the same physical positions at the same timestamps, and only then is it meaningful to estimate the rigid-body transform between the two frames.

A moving average filter was optionally applied to both trajectories to reduce high-frequency jitter before synchronization, which is particularly helpful in radar sequences with bursty noise. After synchronization, the data were passed to the rigid transformation module for spatial alignment.

### 2.4. Trajectory Alignment via Rigid Transformation

Once the radar and camera trajectories were temporally synchronized, the goal was to estimate the rigid-body transformation $T_{rc}=\left[\;R|t\;\right]\in \mathrm{SE}\left(3\right)$, the Special Euclidean group of 3D rigid motions, which represents all rotations and translations in three-dimensional space. This transformation maps 3D points from the radar coordinate frame to the camera frame. This transformation consists of a 3 × 3 rotation matrix R∈SO(3) and a 3 × 1 translation vector $t\in R^{3}$, such that for any radar measurement $p_{r}$, its corresponding point in the camera frame is approximated by:

(8) $$p_{c}\approx R\;p_{r}+t$$

Let ${\left\{p_{r}^{\left(i\right)}\right\}}_{i=1}^{N}$ and ${\left\{p_{c}^{\left(i\right)}\right\}}_{i=1}^{N}$ be the sets of matched 3D points from radar and camera trajectories after time alignment, where *N* is the number of synchronized frames. To compute the optimal transformation, we minimized the Frobenius norm of the residual alignment error using the following objective:

(9) $$\min_{R,t}\;\sum_{i=1}^{N}{∥\;p_{c}^{\left(i\right)}-\left(R\;p_{r}^{\left(i\right)}+t\right)∥}^{2}$$

This classical least-squares problem has a closed-form solution when point correspondences are known and the transformation is rigid (i.e., preserves distances and angles). We applied the following procedure, based on the well-known Kabsch and Umeyama algorithms [27,28]:

1. Compute Centroids

(10) $${\bar{p}}_{r}=\frac{1}{N}\sum_{i=1}^{N}p_{r}^{\left(i\right)},\;{\bar{p}}_{c}=\frac{1}{N}\sum_{i=1}^{N}p_{c}^{\left(i\right)}$$

2. Center the Points

Subtract the centroids from each point:

(11) $${\tilde{p}}_{r}^{\left(i\right)}=p_{r}^{\left(i\right)}-{\bar{p}}_{r},\;{\tilde{p}}_{c}^{\left(i\right)}=p_{c}^{\left(i\right)}-{\bar{p}}_{c}$$

3. Compute Cross-Covariance Matrix

(12) $$H=\sum_{i=1}^{N}{\tilde{p}}_{r}^{\left(i\right)}\;{\tilde{p}}_{c}^{(i)T}$$

4. Compute Optimal Rotation

Apply Singular Value Decomposition (SVD) to $\;H=U\Sigma V^{T}$

Then:

(13) $$R=VU^{T}$$

If det(R)<0, adjust V←V.diag(1,1,−1)

5. Compute Translation

(14) $$t={\bar{p}}_{c}-R\;{\bar{p}}_{r}$$

6. Transformation Output

The final transformation matrix $T_{rc}$ is formed as:

(15) $$T_{rc}=\left[\begin{array}{cc} R & t\\ 0^{T} & 1 \end{array}\right]$$

To improve robustness against outliers, the point sets were preprocessed using RANSAC-based filtering. The RMSE before and after alignment was computed to evaluate the quality of the calibration. This estimated transformation was later validated against the ground-truth transformation measured via the OptiTrack system, as discussed in the next section. This yielded the final transformation $T_{rc}$ that maps radar-based measurements to the camera frame. The correctness and consistency of the transformation were evaluated using two metrics: (1) visual overlay of the aligned trajectories, and (2) RMSE between transformed radar points and the original camera points:

(16) $${\mathrm{RMSE}}_{\mathrm{align}}=\sqrt{\frac{1}{N}\sum_{i=1}^{N}{∥p_{c}^{\left(i\right)}-\left(R_{\;est}\;p_{r}^{\left(i\right)}+t_{\;est}\right)∥}^{2}}$$

Multiple trials using different drone trajectories consistently produced decimeter-level alignment RMSE (approximately 0.12–0.18 m) and sub-degree rotational error (as further validated in the validation section). This confirmed that the trajectory-based rigid alignment method was effective even in the presence of radar noise and partial observation dropout.

### 2.5. Validation Using OptiTrack

To evaluate the accuracy of the estimated radar-to-camera transformation, an OptiTrack motion capture system was employed as an independent ground truth reference. OptiTrack is a marker-based optical tracking platform that uses infrared cameras to detect retroreflective markers in 3D space with high spatial and temporal precision.

In our experimental setup, the OptiTrack system consisted of 4 Flex 3 cameras arranged around a 4 × 4 m indoor laboratory area. The system was calibrated using wand-based dynamic calibration and reference ground plane alignment procedures. Under optimal visibility and lighting conditions, the system achieves sub-millimeter accuracy in position (approximately 0.2–0.3 mm) and angular accuracy within ±0.1 degrees, as reported by the manufacturer [29]. Retroreflective marker clusters were rigidly attached to:

- the camera (with attached markers)
- the radar (with attached markers)
- the Skydio 2 drone

Each sensor has its own coordinate frame: {r} for the radar, {c} for the camera, and {gt} for the global OptiTrack reference. The estimated radar-to-camera transformation $T_{rc}^{est}$ maps radar coordinates to the camera frame, while the ground-truth transformation $T_{rc}^{gt}$ obtained from OptiTrack provides the same mapping in global coordinates. Both are expressed as homogeneous transforms $T_{rc}=\left[\begin{array}{cc} R_{rc} & t_{rc}\\ 0^{T} & 1 \end{array}\right]$, where $R_{rc}$ and $t_{rc}$ denote rotation and translation components, respectively.

The marker constellations were fixed using lightweight rigid mounts to preserve relative geometry and minimize vibration-induced shifts during operation. The static offset between each sensor’s coordinate frame and its attached marker frame was calculated by recording high-frequency stationary data and averaging over time. During drone flights, all three systems—radar, camera, and OptiTrack—recorded 3D trajectories of the Skydio 2 simultaneously. After applying temporal synchronization and rigid transformation estimation, the estimated radar-to-camera transformation $T_{rc}^{est}$ was compared with the reference transformation $T_{rc}^{gt}$ derived from OptiTrack. Alignment accuracy was quantified using the following metrics:

- Translation Error:

(17) $$e_{t}={∥t^{est}-t^{gt}∥}_{2}$$

- Rotation Error (Euler angle-based):

Let $R_{\mathrm{error}}=R^{gt^{-1}}R^{est}$, and extract the axis-angle representation to compute angular deviation.

In addition, the RMS trajectory error after alignment is reported using the RMSE metric defined in Equation (16). These errors were reported across multiple trajectory repetitions, and alignment consistency was verified. The use of OptiTrack ensured a precise and reliable benchmark for quantifying the spatial calibration performance under realistic and repeatable experimental conditions.

This validation step confirmed that the estimated radar-to-camera transformation achieves sub-degree rotational accuracy and decimeter-level translational accuracy (with translation deviations up to approximately 0.27 m), which is acceptable for downstream sensor fusion and robotic applications.

## 3. Experimental Results

All experiments were conducted in an indoor laboratory equipped with an OptiTrack motion-capture system, providing sub-millimeter positional accuracy at 240 Hz. The sensor suite consisted the radar and the camera, mounted on separate tripods approximately 2 m apart with non-parallel orientations. A Skydio 2 drone was used as the dynamic target, performing smooth and repeatable flight trajectories within the shared field of view of both sensors.

Two experimental scenarios were evaluated:

1. **Calibration Scene**—a forward-and-reverse loop trajectory designed to provide sufficient spatial coverage for extrinsic parameter estimation.
2. **Validation Scene**—an independent arc-shaped trajectory used to test the generalization of the estimated calibration parameters to unseen motion.

Data from radar, camera, and OptiTrack were recorded simultaneously for each scenario, enabling both the estimation and verification of radar–camera extrinsic calibration.

### 3.1. Calibration Scene

To evaluate the robustness and generalizability of the proposed targetless radar–camera calibration pipeline, experiments were conducted using multiple flight trajectories and sensor configurations. First, a calibration experiment was performed, in which the mmWave radar and the RGB-D camera were placed at fixed but non-parallel orientations to simulate realistic deployment conditions. The drone executed a forward–reverse trajectory to provide sufficient coverage for accurate extrinsic calibration. Subsequently, an independent validation flight was recorded without re-calibrating the system, in which the drone followed a different trajectory. This additional experiment was used to assess how well the estimated transformation generalized to unseen motion sequences. In both cases, a high-precision OptiTrack motion capture system served as the ground truth reference for evaluating spatial alignment accuracy.

**Figure 2:**
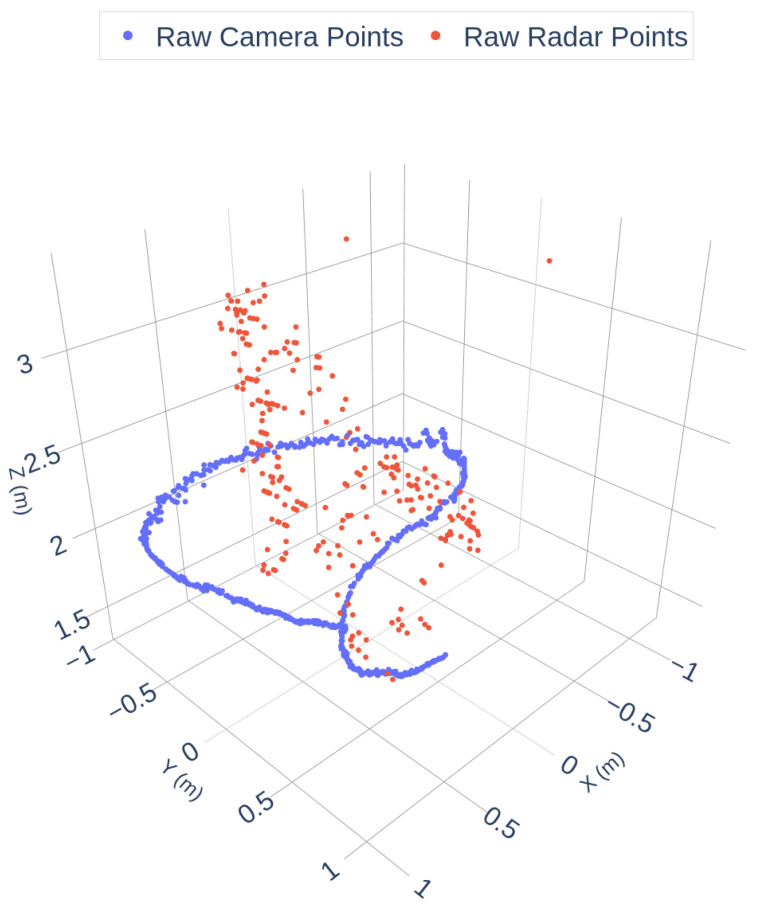
Raw Camera and Radar Trajectories.

**Figure 3:**
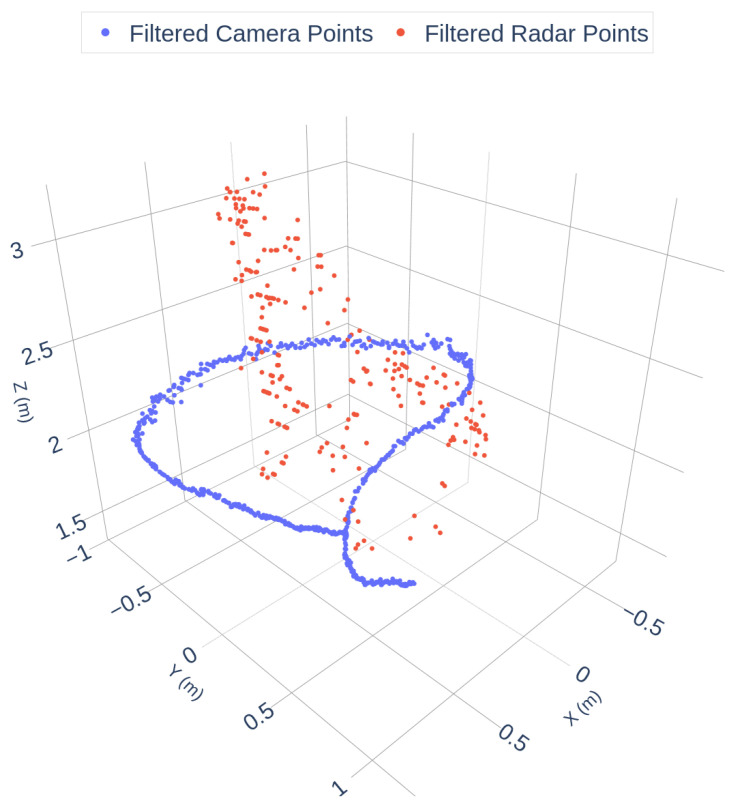
Filtered Camera and Radar Trajectories.

As shown in Figure 2, the radar data exhibits significant sparsity and angular noise, while the camera trajectory is dense and smooth. To enhance data quality, a filtering pipeline was applied, including geometric constraints, DBSCAN clustering, and RANSAC outlier removal. The filtered results are shown in Figure 3:

After preprocessing, 670 inlier points were retained from the camera and 235 from the radar. To achieve temporal alignment, radar trajectories were shifted iteratively in 20 ms increments over a ±2-s window. The RMSE was computed for each offset, and the minimum was found at +0.460 s, which was applied to synchronize the radar data with the camera timeline. As discussed in the temporal synchronization subsection, such a temporal offset naturally induces a corresponding spatial shift along the drone’s motion path, since the radar samples effectively represent an earlier or later position of the platform. Correcting Δt is therefore essential for eliminating this systematic trajectory discrepancy prior to extrinsic alignment.

**Figure 4:**
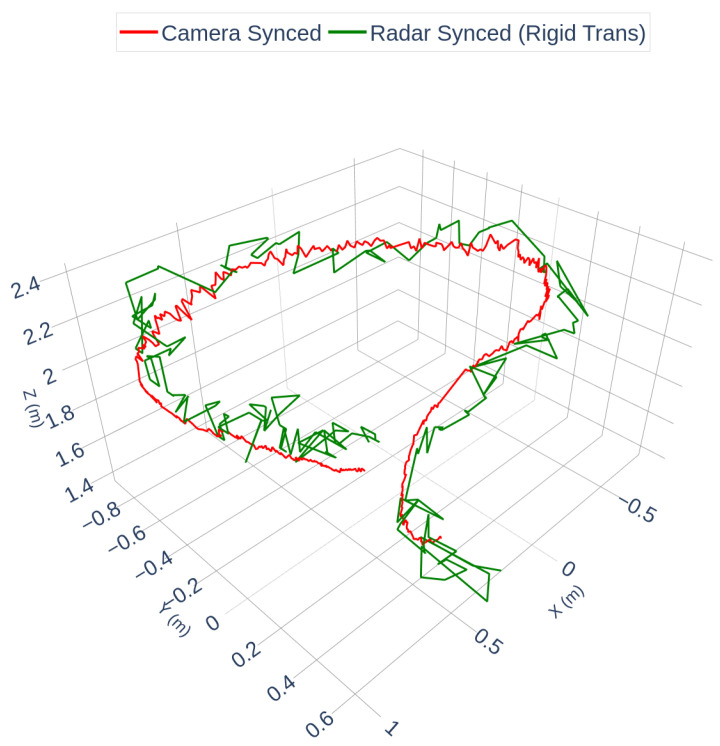
Synchronized camera and rigid-transformed radar trajectories (discrete points). The radar trajectory has been shifted by the estimated time offset Δt=0.46 s and mapped into the camera frame using the Kabsch/Umeyama solution. This figure shows the actual calibration result used for RMSE computation.

**Figure 5:**
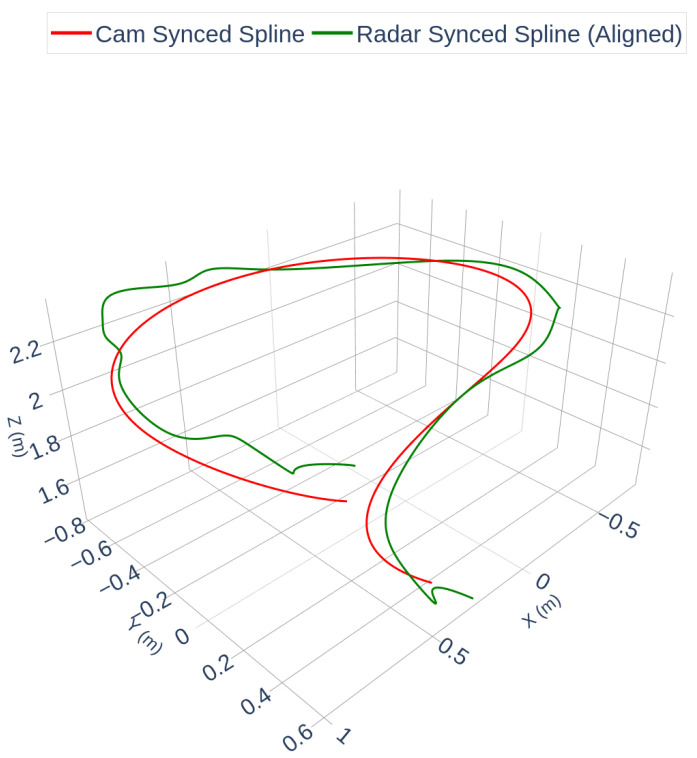
Spline-interpolated visualization of the aligned trajectories from Figure 4. Cubic spline interpolation is applied only for visual clarity and does not alter the estimated extrinsic parameters or the alignment error.

Following synchronization, a rigid transformation was computed using the Kabsch/Umeyama algorithm to estimate the extrinsic calibration between the radar and camera frames. Figure 4 visualizes the resulting alignment using the discrete synchronized point trajectories. Figure 5 shows a spline-interpolated version of the same aligned trajectories, included solely to improve visual clarity in curved segments; the spline operation is not part of the calibration and does not affect the estimated extrinsic parameters or RMSE.

Figure 4 and Figure 5 illustrate that the radar trajectory closely matches the camera trajectory after temporal synchronization and rigid spatial alignment, with some minor deviations in sparse segments. The final alignment RMSE between the discrete trajectories in Figure 4 was 0.1582 m. To assess calibration accuracy, the estimated transformation was compared against OptiTrack ground truth. The results are summarized in Table 1.

These results confirm that the proposed targetless calibration pipeline performs reliably under real-world sensor noise and low-reflectivity conditions. The alignment remained accurate both geometrically and temporally, validating the method’s suitability for radar–camera extrinsic estimation in robotics applications, particularly in scenarios requiring precise spatial correspondence between heterogeneous sensors. Furthermore, the robustness of the approach under sparse radar measurements and measurement bias highlights its potential for autonomous systems operating in cluttered or dynamically changing environments, where traditional calibration targets are impractical or unavailable.

### 3.2. Cross-Trajectory Validation

To further evaluate the robustness and generalizability of the proposed calibration pipeline, the estimated transformation parameters derived from the first scene were applied to an independent drone flight sequence. In this experiment, no additional recalibration or parameter optimization was performed. Instead, the rotation matrix *R* and translation vector *t* computed during the initial calibration were directly used to transform the new radar trajectory into the camera reference frame. The validation dataset comprised radar and RGB-D camera measurements acquired while the drone followed a smooth arc-shaped flight path distinct from the original calibration motion. Raw and filtered point clouds were processed using the same DBSCAN clustering and RANSAC filtering pipeline described previously. After temporal alignment via spline-based timestamp interpolation, the radar and camera trajectories were compared against the OptiTrack ground truth to quantify spatial consistency.

The cross-scene evaluation demonstrated that the proposed calibration maintained decimeter-level alignment accuracy (with RMSE around 0.12–0.17 m) without the need for re-optimization, confirming its transferability across varying flight paths and motion dynamics. This consistency across datasets further indicates that the estimated extrinsic parameters capture a physically meaningful transformation between the radar and camera coordinate frames rather than scene-dependent fitting. Additionally, the method exhibited resilience to radar sparsity and measurement bias, underscoring its potential for real-time deployment in autonomous platforms where environmental variations and sensor noise are unavoidable.

**Figure 6:**
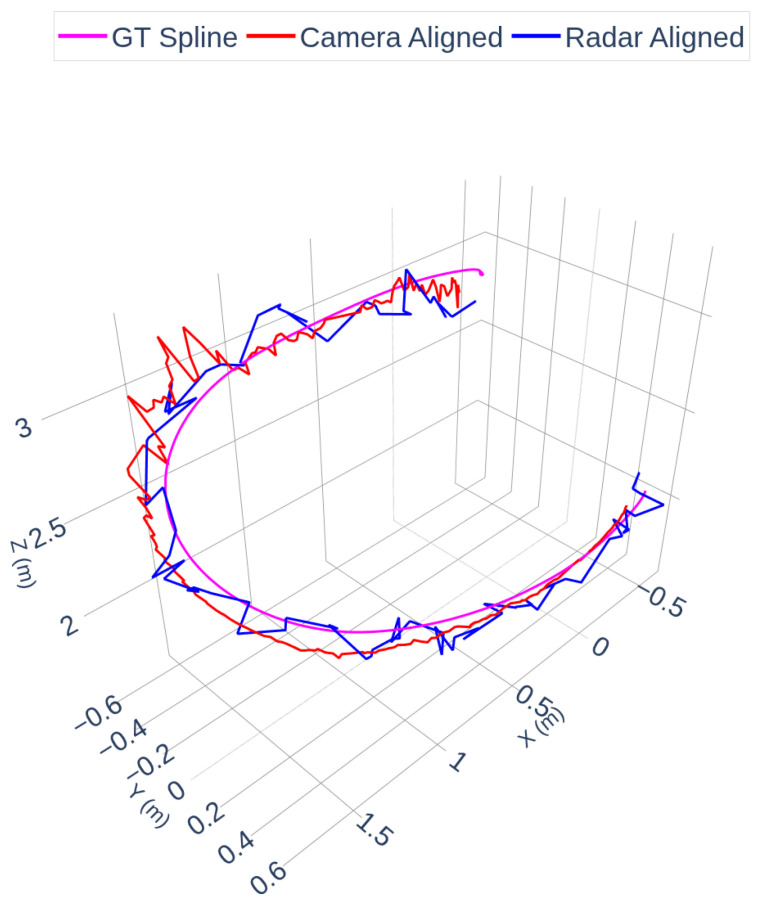
Camera, Radar and OptiTrack Trajectories. Note: “GT Spline” denotes the cubic spline interpolation of the OptiTrack ground-truth trajectory for visualization and temporal resampling purposes.

Figure 6 shows the ground truth trajectory spline (magenta), together with the camera-aligned spline (red) and the radar-aligned spline (blue). The overlay demonstrates close agreement in the global shape and position of the trajectories across modalities. The radar trajectory exhibits higher local variance due to sparser detections, which is consistent with observations in Scene 1.

In these figures, the term “GT Spline” refers to the cubic spline interpolation of the OptiTrack ground-truth trajectory. The OptiTrack system provides high-frequency (240 Hz) marker positions, which are resampled via spline interpolation to create a smooth, continuous reference curve used for timestamp alignment and visual comparison. This interpolation does not alter the ground-truth geometry; it only provides a continuous representation of the measured trajectory.

**Figure 7:**
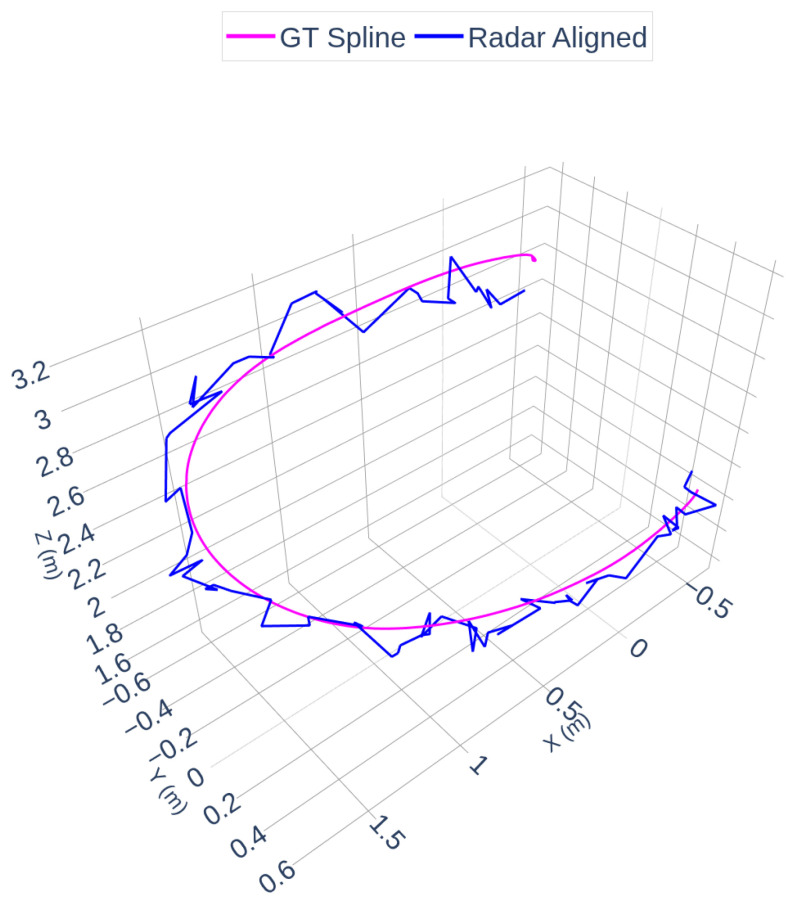
Radar and OptiTrack Trajectories.

**Figure 8:**
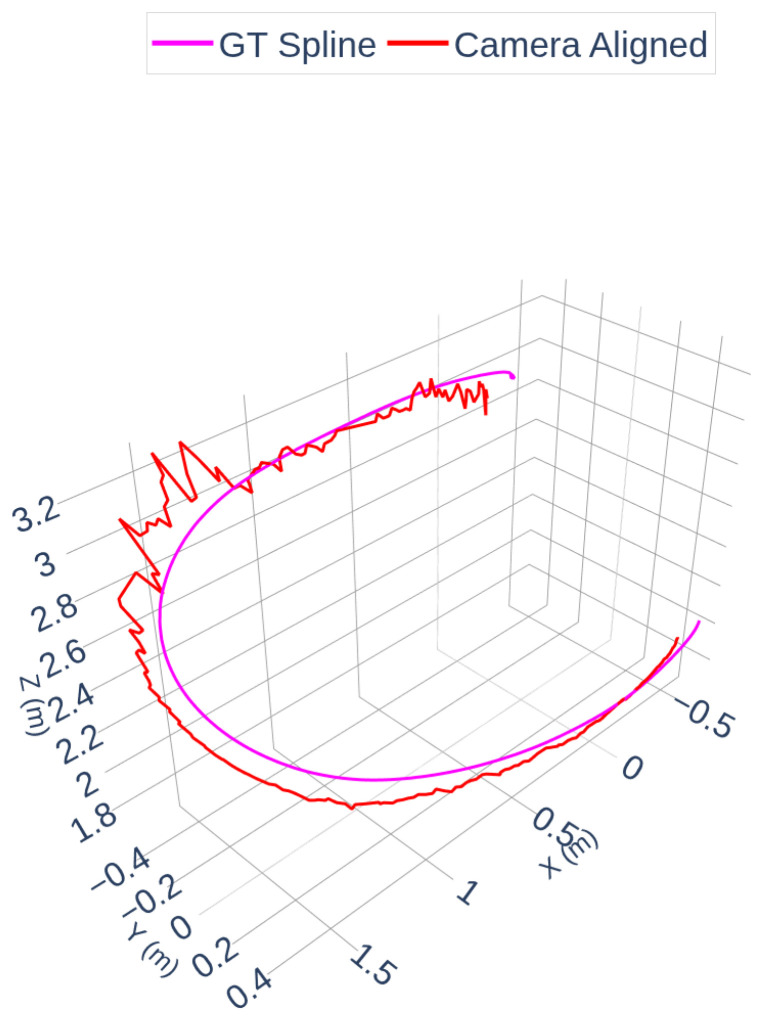
Camera and OptiTrack Trajectories.

Figure 7 and Figure 8 illustrate the radar and camera aligned trajectories separately compared to the OptiTrack spline. In Figure 7, the radar-aligned trajectory closely follows the ground truth path, with minor deviations around curved segments of the flight. Figure 8 highlights the camera-aligned trajectory, which shows similar consistency but exhibits slightly increased jitter due to the raw depth estimation noise in certain parts of the motion.

The accuracy of the spatial alignment was quantified using Root Mean Square Error (RMSE) computed between the spline-interpolated radar trajectory and the ground truth spline. The RMSE after spline timestamp interpolation alignment was 0.1171 m, indicating decimeter-level spatial agreement (approximately 12 cm RMSE) in the aggregate path reconstruction. Additionally, the radar spline timestamp interpolation RMSE was measured as 0.1697 m, reflecting the sparser nature of the radar point cloud.

These results confirm that the estimated extrinsic parameters retained high alignment performance when applied to an independent motion sequence, without any recalibration or additional parameter tuning. The consistency of the RMSE metrics across both experiments demonstrates the practical transferability of the proposed calibration approach under real-world deployment conditions.

### 3.3. Failure Cases and Limitations

Although the proposed calibration pipeline performs reliably under normal experimental conditions, several failure cases were observed or theoretically identified. These scenarios highlight the inherent limitations of trajectory-based targetless calibration and provide guidance for practical deployment.

(1) Straight-line motion.

If the drone moves along a near-straight trajectory with minimal rotational excitation, the spatial alignment problem becomes degenerate because multiple rigid transformations can produce equally low RMSE. In such cases, the rotation around the motion axis is weakly constrained. In our experiments, slight curvature in the flight path prevented full degeneracy, but the sensitivity to motion geometry remains a known limitation of trajectory-based methods.

(2) Limited orientation coverage.

Trajectories that do not contain sufficient variations in heading or altitude reduce the observability of the full 6-DoF extrinsic parameters. When the trajectory spans only a narrow portion of the flight volume, the cross-covariance matrix becomes ill-conditioned, increasing translation uncertainty. This effect was visible in sparse radar segments where local curvature was low.

(3) Sharp radar maneuvers and Doppler bursts.

Rapid drone acceleration or turns occasionally produced radar point clouds with unstable clustering behavior. During these intervals, DBSCAN either detected multiple small clusters or failed to isolate a consistent target. Such frames were automatically down-weighted or rejected by RANSAC, but prolonged high-maneuver phases can reduce accuracy due to intermittent radar sparsity.

(4) YOLOv5 detection dropouts.

Temporary loss of visual detections occurred when the drone rotated sideways, causing partial occlusions and low confidence bounding boxes. In these cases, 3D depth estimation became unreliable. The smoothing filter (Equation (1)) reduced short-term jitter, but longer YOLO dropouts propagate as gaps in the camera trajectory and limit the number of valid correspondences.

(5) Radar sparsity and range bias.

The radar occasionally produced fewer than 5 points per frame due to low radar cross-section. While fallback centroid estimation ensured continuity, these frames contributed higher residual error. Furthermore, a consistent ~25 cm range overestimation introduces a systematic translation bias that rigid alignment cannot fully eliminate.

(6) Temporal synchronization sensitivity.

If the motion profile contains large stationary intervals, the RMSE surface in Equation (6) becomes flatter, reducing the ability to uniquely determine Δt. Although this was not observed in our trajectories, stationary motion is a known failure mode of passive synchronization.

These limitations are common to targetless calibration approaches relying on natural motion rather than dedicated calibration patterns. Nevertheless, the proposed method remained robust under realistic indoor conditions and achieved consistent decimeter-level accuracy across multiple trajectories.

### 3.4. Analysis and Discussion

The experimental results demonstrated the efficacy and robustness of the proposed targetless radar-camera calibration method, even under challenging operational conditions. Despite inherent radar data sparsity and dynamically complex drone trajectories, the method achieved consistent spatial alignment, as indicated by the low RMSE values (below 18 cm in both scenarios).

It is important to emphasize that temporal synchronization and spatial calibration are two separate steps: the former aligns the radar and camera measurements in time, while the latter estimates the rigid transform between their coordinate frames. The trajectories in Figure 4 represent the result after both steps, whereas Figure 5 is a smoothed visualization of the same aligned data.

Minor discrepancies in rotation and translation when compared with OptiTrack measurements were noted, primarily attributed to systematic radar errors—most notably, a consistent distance overestimation (~25 cm). Nevertheless, these deviations were within acceptable operational tolerances. Euler angle differences remained minimal, reflecting strong alignment accuracy and indicating that the method is particularly reliable for angular alignment.

The presented methodology shows considerable promise for real-world autonomous navigation systems, where ease of use, robustness in dynamic environments, and reliable integration of multiple sensor modalities are crucial. Future enhancements may include advanced radar preprocessing techniques, improved temporal synchronization methods, and testing in outdoor environments to further validate and extend the applicability of this calibration approach.

As summarized in Table 2, the proposed method achieves the lowest rotational error among the compared targetless radar–camera calibration approaches, with an $\ell_{2}$ rotation norm of 0.70°, improving on the 1.73°–7.10° range reported in [12,16,18]. The RPE/RMSE of 0.158 m is also comparable to or better than prior work, even though our evaluation is performed on a single unconstrained drone trajectory rather than repeated structured automotive sequences. The main weakness is the larger translation norm (274.9 mm), which is consistent with the approximately 25 cm positive range bias observed in the radar measurements and the known 2–5% depth scale error of the RGB-D camera; these systematic effects dominate the absolute extrinsic mismatch while leaving the relative trajectory alignment (and hence the RMSE) at the decimeter level.

Beyond these classical geometric methods, recent targetless radar–camera calibration studies also explore deep learning-based pipelines and report sub-degree rotational accuracy and translation errors typically below 10 cm under structured driving scenarios. For example, Luu et al. [34] introduce an end-to-end deep network for automatic extrinsic calibration and achieve rotation errors in the range of 0.5°–0.9° with translation errors of 5–10 cm. Similarly, Liu et al. [35] propose a track-to-track association framework and report average rotation and translation errors of 0.814° and 0.075 m, respectively. While these approaches demonstrate strong performance on automotive datasets with dense radar tensors and consistent motion patterns, they rely heavily on supervised learning and high-quality radar returns. In contrast, the method proposed in this work achieves comparable sub-degree rotational accuracy under significantly more challenging conditions characterized by extremely sparse mmWave returns (3–10 points per frame), low-RCS aerial targets, and unconstrained six-degrees-of-freedom (6-DoF) motion. Despite these difficulties, our pipeline maintains decimeter-level translation accuracy without requiring training data or specialized calibration targets, underscoring its suitability for practical deployment in robotics applications where dense radar observations or large annotated datasets are unavailable.

### 3.5. Parameter Sensitivity Analysis

The proposed pipeline includes several parameters that influence the robustness of clustering, outlier removal, and smoothing. Although the method performed consistently across all experiments, it is important to outline how these parameters affect performance and how their values were selected.

(1) DBSCAN parameters (ε, MinPts).

The DBSCAN radius ε=0.5 m was chosen to reflect the typical spread of radar returns from the drone at a 3–5 m range. Smaller values (<0.3 m) tended to fragment the radar points into multiple clusters, causing intermittent loss of the target and reducing trajectory continuity. Larger values (>0.7 m) merged background noise into the main cluster, leading to biased centroid estimates. The parameter MinPts = 3 served as a balance between rejecting single-frame spurious detections and retaining valid but sparse radar returns.

(2) RANSAC outlier threshold (τ).

The RANSAC residual threshold τ=0.3 m rejected points that exceeded the expected motion smoothness of the drone. Tighter thresholds (<0.2 m) removed too many valid radar samples, especially in curved trajectories where motion changes rapidly. Looser thresholds (>0.4 m) allowed noisy radar outliers to pass, increasing alignment RMSE by 2–4 cm. Thus, τ=0.3 m provided a robust compromise that preserved trajectory shape while filtering radar-induced noise.

(3) Smoothing window size (*w*).

The moving-average window w=5 was selected to suppress jitter in depth-based 3D estimates without blurring rapid motion. Smaller windows (w=1–3) left residual jitter that degraded temporal alignment and increased RMSE. Larger windows (w≥7) oversmoothed sharp turns and reduced the observability of orientation changes, slightly weakening the estimation of rotation parameters. Overall, w=5 preserved geometric fidelity while stabilizing the trajectories.

(4) Time offset search range.

The grid search range of ±2 s with a 10 ms step ensured that the global RMSE minimum was consistently detected. Smaller ranges risked missing the true delay; coarser steps increased temporal jitter after alignment. The chosen values ensured stable synchronization without overfitting.

Across all experiments, these parameters were found to be stable and insensitive to small perturbations. Varying each parameter within a reasonable range affected the RMSE by only a few centimeters and did not alter the overall calibration outcome. This confirms that the method is robust to parameter selection and behaves consistently under realistic noise conditions.

## 4. Conclusions

This study successfully demonstrated a robust and effective targetless radar–camera calibration method based on trajectory alignment via rigid transformations. The proposed approach was validated through comprehensive experiments involving a dynamically maneuvering Skydio 2 drone equipped with retroreflective markers for high-precision ground-truth tracking.

The experiments confirmed that despite significant radar signal attenuation, sparse detections, and dynamically complex motion, the calibration pipeline reliably aligned radar and camera measurements with minimal residual error in both spatial and temporal domains. Quantitative analysis against the OptiTrack reference system showed that the proposed method achieves sub-degree rotational accuracy with rotation errors of 0.68°, 0.09°, and 0.15° along the three axes. The translational difference between the estimated extrinsic parameters and the OptiTrack ground truth was measured as Δt=[0.25706,0.06930,−0.06850] m, corresponding to an $L_{2}$ norm of 0.275 m. These values represent the absolute mismatch between the estimated radar–camera transform and the independently measured reference.

A closer inspection of the per-axis components shows that the discrepancy is dominated by systematic range-related biases in both sensors. The depth camera introduces a well-known 2–5% depth offset (approximately 0.15–0.30 m within the 3–6 m operating range). Likewise, the radar exhibits a consistent ~25 cm positive range bias under low-RCS conditions. These sensor-induced effects together explain the full translational mismatch of 0.275 m, while the alignment RMSE of 0.11–0.17 m demonstrates that the trajectory-alignment pipeline itself is stable and repeatable even when individual sensors exhibit geometric distortion or range bias.

In comparison with existing targetless radar–camera calibration methods, our approach achieves sub-degree rotational accuracy and decimeter-level translation errors that are competitive with or better than prior work, as summarized in Table 2. Importantly, these results are obtained under substantially more challenging conditions—extremely sparse radar returns, a low-RCS aerial target, and fully 6-DoF UAV motion—without relying on learned models, dense automotive radar tensors, or large annotated datasets. This highlights the practicality of the proposed trajectory-alignment pipeline for real-world robotic platforms where dense radar observations or extensive calibration data are not available.

Overall, the findings validate the real-world applicability of the method for multi-sensor fusion scenarios requiring reliable calibration without dedicated targets or carefully controlled environments. Accurate radar–camera extrinsics are essential for applications such as autonomous navigation, SLAM, and perception-driven control. Future work will explore integrating this calibration framework into aerial robots trained via reinforcement learning, where precise sensor alignment is critical for safe and effective multi-modal perception. 

## Figures and Tables

**Table 1:** Quantitative Evaluation vs. OptiTrack Ground Truth.

Metric	Estimated Value	OptiTrack Ground Truth	Deviation
Rotation (Euler, deg)	Roll: 7.25°, Pitch: 54.30°, Yaw: 8.12°	Roll: 7.93°, Pitch: 54.21°, Yaw: 8.27°	0.68°/0.09°/0.15°
Translation (m)	[−0.68394,−0.068698,1.7805]	[−0.941,−0.138,1.849]	$L_{2}$ norm = 0.275 m
RMSE After Alignment (m)	0.1582	—	—

**Table 2:** Detailed quantitative comparison of targetless radar–camera calibration methods. Rotation errors are reported per Euler axis (γ,θ,φ) and as $\ell_{2}$ norm. Translation errors are given per axis (X,Y,Z) in millimeters and as $\ell_{2}$ norm. RPE/RMSE values are in meters.

Method	γ (°)	θ (°)	φ (°)	Rot. Norm (°)	*X* (mm)	*Y* (mm)	*Z* (mm)	Trans. Norm (mm)	RPE/RMSE (m)
Schöller et al. [12]	1.970	2.080	5.057	5.81	–	–	–	–	0.039
Jin et al. [16]	1.269	4.048	1.801	4.61	–	–	–	–	0.186
Du et al. [17]	4.470	1.235	5.377	7.10	4.300	7.000	23.061	24.48	0.127
Zhu et al. [18]	1.106	0.976	0.904	1.73	12.674	4.100	9.706	16.48	0.026
Proposed (ours)	0.680	0.090	0.150	0.700	257.060	69.300	−68.500	274.90	0.158

Note: The relatively larger translation norm in the proposed method is consistent with an approximately 25 cm systematic positive range bias observed in the radar measurements, which shifts the radar-derived trajectory outward in the radial direction while leaving rotation estimation effectively unaffected.

## Data Availability

The raw data supporting the conclusions of this article will be made available by the authors on request.
